# Impact of ultrasound cycloplasty on ocular biomechanics and refractive parameters: a comprehensive review

**DOI:** 10.3389/fmed.2026.1783195

**Published:** 2026-02-17

**Authors:** Liping Yang, Jinli Gao, Yuhui Yan, Guitao Shi

**Affiliations:** Department of Ophthalmology, The Affiliated Hospital of Inner Mongolia Medical University, Hohhot, China

**Keywords:** anterior segment, glaucoma, ocular biomechanics, refractive parameters, ultrasound cycloplasty

## Abstract

Ultrasound cycloplasty (UCP) is a minimally invasive, non-incisional glaucoma treatment that lowers intraocular pressure through high-intensity focused ultrasound–mediated cyclodestruction. Although UCP has been increasingly adopted in clinical practice, and multiple studies have reported its efficacy and safety, evidence regarding its effects on ocular biomechanics and refractive parameters remains limited, leaving critical knowledge gaps in understanding postoperative structural and functional outcomes. This review focuses on these underexplored aspects, synthesizing available clinical and translational studies to identify current limitations and highlight areas requiring further investigation. Overall, existing evidence suggests that UCP achieves sustained intraocular pressure reduction with minimal long-term impact on ocular biomechanics or visual function. By critically integrating these findings, the review aims to fill knowledge gaps, provide insight for clinicians and researchers, and guide optimized patient selection and postoperative monitoring.

## 1 Introduction

Glaucoma remains a leading cause of irreversible blindness worldwide, with elevated intraocular pressure (IOP) representing the primary modifiable risk factor (1). When medical therapy or conventional filtering surgery fails or is contraindicated, cyclodestructive procedures targeting the ciliary body become important therapeutic alternatives (2). Ultrasound cycloplasty (UCP) is a non-incisional technique that employs high-intensity focused ultrasound (HIFU) to selectively ablate the ciliary epithelium, thereby reducing aqueous humor production while preserving adjacent ocular structures (3). Compared with traditional transscleral cyclophotocoagulation (TSCPC), UCP provides superior spatial selectivity, standardized energy delivery, and improved reproducibility, resulting in a more favorable safety and tolerability profile (4).

Over the past decade, major advances in UCP research have established its efficacy in lowering IOP and reducing medication burden across a range of glaucoma subtypes. However, most clinical studies to date have focused on pressure-centric endpoints, whereas the broader biomechanical and refractive consequences of UCP remain comparatively underexplored. This is increasingly relevant, as glaucoma interventions can influence corneal curvature, anterior segment geometry, and tissue mechanical behavior, all of which may affect visual quality and long-term ocular stability (5). Although isolated reports have described postoperative changes in corneal astigmatism or anterior segment parameters following UCP, findings remain heterogeneous, fragmented, and often secondary to primary efficacy outcomes. The field currently lacks an integrated synthesis linking these clinical observations to biomechanical mechanisms. Accordingly, this Review critically evaluates existing evidence on the impact of UCP on ocular biomechanics and refractive parameters, identifies current debates and knowledge gaps, and highlights priorities for future research.

## 2 Overview of ultrasound cycloplasty

### 2.1 Development and rationale

Cyclodestructive approaches have long been used in refractory glaucoma to suppress aqueous humor production via ciliary body ablation. Conventional modalities such as TSCPC are limited by variable energy delivery, collateral tissue damage, and unpredictable clinical outcomes (6).

The introduction of HIFU technology represented a major advance, enabling precise, depth-controlled energy deposition within the ciliary body. UCP emerged from this development as a non-incisional technique capable of inducing localized coagulative necrosis of the ciliary epithelium while sparing the sclera, lens, and retina (3). Multicenter clinical studies consistently report mean IOP reductions of approximately 30–40% at 12 months, with a low incidence of vision-threatening complications, positioning UCP as a viable option for patients unsuitable for incisional surgery or with prior surgical failure (7, 8).

### 2.2 Surgical technique and imaging assessment

UCP is typically performed under topical or peribulbar anesthesia using a circular probe designed to align treatment sectors with the anatomical location of the ciliary body. Energy delivery parameters are standardized but may be adjusted according to axial length and anterior chamber configuration (9).

Ultrasound biomicroscopy (UBM) and anterior segment optical coherence tomography (AS-OCT) constitute the principal imaging modalities used to assess postoperative anatomical changes. These tools have enabled detailed characterization of UCP-induced modifications in anterior chamber depth, angle configuration, and ciliary body morphology (10).

### 2.3 Indications and patient selection

UCP is most commonly applied in primary open-angle glaucoma, pseudoexfoliative glaucoma, and refractory glaucoma following failed filtering or drainage procedures. Its non-incisional nature is particularly advantageous in patients with thin sclera, ocular surface disease, or high surgical risk (11).

Current debate persists regarding the role of UCP in earlier-stage disease. While emerging studies suggest potential benefit in selected cases of moderate glaucoma, careful patient selection remains essential, particularly in eyes with advanced optic nerve damage or very low baseline IOP, where pressure-independent mechanisms may dominate visual decline (12).

### 2.4 Clinical performance and safety

Prospective and retrospective clinical series consistently demonstrate sustained and clinically meaningful IOP reduction following UCP. Meta-analyses confirm significant postoperative IOP lowering and reduced dependence on topical medications, with adverse events predominantly mild and transient (8).

Although rare postoperative complications—including transient hypotony, anterior chamber inflammation, and isolated neurotrophic keratopathy—have been reported, these events generally resolve with conservative management (13). Collectively, these findings support UCP as a relatively safe cyclodestructive modality when appropriate patient selection and postoperative monitoring are employed.

## 3 Impact on anterior segment

### 3.1 Anterior chamber depth and angle configuration

A growing body of evidence indicates that UCP induces measurable changes in anterior chamber morphology, particularly in eyes with angle-closure mechanisms. Imaging studies consistently demonstrate increases in central anterior chamber depth and anterior chamber area following UCP, suggesting partial relief of anterior segment crowding and stabilization of angle configuration (14). This interpretation is supported by quantitative anterior segment imaging data from prospective UCP cohorts showing statistically significant increases in ACD, anterior chamber area (ACAr), and anterior chamber angle (ACAn) when compared with preoperative values, reinforcing the notion that UCP can induce structural expansion of the anterior chamber in addition to IOP reduction (UBM analysis revealed increased ACD, enlarged pupillary diameter, expanded ACAr and widened ACAn after UCP, all *p* < 0.05) (7). These observations represent a recent advance in understanding the structural effects of UCP and indicate that its impact extends beyond simple pressure lowering to include measurable anterior segment expansion in select glaucoma subtypes.

### 3.2 Ciliary body and zonular interactions

Recent clinical imaging studies further suggest that UCP induces measurable changes in ciliary body–related structures, with potential implications for zonular tension, anterior segment biomechanics, and postoperative refractive stability. Wang et al. reported that zonular length decreased significantly across multiple quadrants after UCP, indicating localized modulation of lens-supporting structures. Ciliary muscle thickness (CMT) increased transiently in the early postoperative period, consistent with tissue remodeling or reversible edema (15).

These structural changes were accompanied by alterations in the trabecular–ciliary process angle (TCA), with the temporal quadrant decreasing from 37.4° ± 2.1° preoperatively to 34.8° ± 1.9° at 3 months, reflecting subtle shifts in ciliary body positioning and zonular tension. Complementary observations by Liu et al. demonstrated postoperative enlargement of anterior chamber area and minor adjustments in lens vault, further supporting the concept that UCP influences the biomechanical balance of the anterior segment rather than acting solely through aqueous suppression (14).

## 4 Impact on refractive parameters

### 4.1 Corneal curvature and astigmatic changes

Available clinical evidence indicates that UCP induces only minimal and predominantly transient changes in corneal curvature and astigmatism. In a prospective multicenter cohort, mean induced corneal astigmatism peaked at 0.88 D at 1 month and 1.16 D at 6 months, whereas total refractive astigmatism remained lower, suggesting that early postoperative astigmatic shifts were primarily corneal in origin (16). Importantly, most eyes exhibited stabilization of both corneal curvature and total astigmatism by intermediate follow-up, with best-corrected visual acuity (BCVA) returning toward baseline, underscoring the transient and clinically limited nature of these refractive changes (17).

This pattern is reinforced by longitudinal cohort data showing statistically significant early changes in anterior and posterior keratometric parameters at 1 week after UCP, followed by gradual normalization by 3–12 months. In that study, mean anterior astigmatism increased acutely from ~1.12 D preoperatively to ~2.17 D at 1 week, then progressively declined, with no significant difference from baseline at 12 months (18). Taken together, these findings suggest that although UCP may induce small, early alterations in corneal curvature and anterior segment astigmatism, such effects are generally mild, self-limited, and unlikely to result in sustained refractive instability, consistent with the non-incisional nature of UCP and its limited perturbation of corneal biomechanical equilibrium.

### 4.2 Spherical equivalent and visual outcomes

Clinical evidence consistently indicates that UCP preserves refractive stability and visual function while achieving effective IOP reduction. In a 12-month prospective cohort of eyes undergoing UCP after failed glaucoma surgery, BCVA remained stable despite substantial IOP lowering, with minimal change in spherical equivalent (19). These observations are corroborated by a comprehensive meta-analysis demonstrating that, although UCP significantly reduces IOP, BCVA neither improves nor worsens to a statistically significant degree overall (8).

Intermediate-term cohort studies in primary angle-closure glaucoma similarly report stable visual acuity over follow-up approaching 2 years, indicating that clinically meaningful shifts in spherical equivalent refractive error are uncommon (20). Further prospective evidence in eyes with preserved functional vision showed that mean BCVA remained essentially unchanged through 12 months, with only 2.6% of patients experiencing a decline greater than two lines, highlighting the favorable optical safety profile of UCP (21). Collectively, these data support the conclusion that UCP does not induce clinically meaningful changes in spherical equivalent or visual acuity in the majority of treated eyes, thereby preserving optical quality alongside sustained IOP control.

## 5 Potential mechanisms linking UCP to biomechanical and refractive changes

### 5.1 Tissue remodeling after focused ultrasound delivery

HIFU used in UCP delivers precisely localized thermal energy to the ciliary epithelium, producing coagulative necrosis of target tissue while largely sparing adjacent muscular and connective structures. Animal histological studies demonstrate segmental necrosis confined to the ciliary processes without observable damage to the sclera or lens, confirming that thermal effects are spatially restricted (22).

Beyond ophthalmic applications, HIFU has been extensively studied in other soft tissues, where it has been shown to induce collagen denaturation followed by collagen reorganization and neosynthesis, as well as alterations in elastin architecture, through temperature-dependent modulation of extracellular matrix–related signaling and wound-healing pathways (23). These processes are thought to reflect a controlled reparative response rather than indiscriminate tissue destruction, resulting in localized ECM remodeling within the treated volume. Taken together, evidence from ocular histology and non-ocular HIFU models supports the concept that UCP induces a controlled and localized tissue remodeling response at the level of the ciliary body, sufficient to reduce aqueous humor production while avoiding widespread biomechanical disruption of surrounding non-target ocular tissues.

### 5.2 Altered aqueous dynamics and structural balance

By suppressing aqueous humor production, UCP induces clinically significant IOP reduction, thereby altering the mechanical load across the corneoscleral shell. Importantly, changes in IOP alone have been shown to modulate corneal biomechanical properties independent of direct tissue injury. In patients with primary open-angle glaucoma, controlled IOP reduction was associated with significant decreases in corneal stiffness and Young’s modulus, with stiffness declining from ~88.6 N/m to ~67.2 N/m and modulus from ~0.71 MPa to ~0.54 MPa (all *p* < 0.001), demonstrating adaptive biomechanical responses to a lower pressure environment (24).

Experimental OCT-based elastography models further show that variations in applied IOP significantly influence corneal wave propagation and estimated Young’s modulus, underscoring the tight coupling between fluid dynamics and tissue mechanics (25). Physiological studies of corneal viscoelasticity similarly demonstrate that baseline IOP modulates corneal viscoelastic parameters within normal pressure ranges (26). Collectively, these findings support an indirect mechanism whereby IOP lowering after UCP alters tissue loading and deformation behavior, providing a biomechanical explanation for transient anterior segment and refractive changes observed clinically.

### 5.3 Inflammatory responses and healing

Although UCP is generally well tolerated, postoperative biochemical changes within the anterior segment microenvironment have been documented. Aqueous humor analyses reveal that inflammatory mediators such as MCP-1 and TGF-β1 are elevated in patients with poorer surgical outcomes, suggesting that cytokine profiles may reflect individual healing trajectories and influence efficacy (27).

In broader ocular disease contexts, anterior chamber inflammation has been associated with reductions in corneal hysteresis and corneal resistance factor, indicating that inflammatory states can modulate ocular biomechanical behavior (28). Although postoperative inflammation after UCP is typically mild and transient, these data suggest that early biochemical healing responses may temporarily influence extracellular matrix composition and viscoelastic properties. Consistent with this interpretation, prospective UCP cohorts report mild anterior chamber reactions in a minority of patients, all resolving without long-term sequelae, reinforcing that inflammatory responses do not generally translate into durable biomechanical dysfunction or refractive instability.

### 5.4 Comparison with other cyclodestructive procedures

UCP, continuous-wave cyclophotocoagulation (CW-CPC), and micropulse transscleral cyclophotocoagulation (MP-CPC) share the common therapeutic objective of reducing aqueous humor production; however, they differ substantially in energy delivery mechanisms, tissue selectivity, and downstream biomechanical impact (29). Laser-based CPC relies on photothermal absorption by pigmented tissues and is inherently associated with broader heat diffusion, which may provoke diffuse inflammation and collateral scleral injury. In contrast, UCP delivers circumferential, spatially confined high-intensity focused ultrasound, producing segmental ablation of the ciliary processes while largely preserving adjacent scleral and limbal structures.

Comparative clinical data indicate that UCP and MP-CPC achieve similar mid-term IOP reduction, but differ in postoperative inflammatory burden and complication profiles. In a retrospective comparative study, both modalities produced significant IOP lowering at 6–12 months; however, MP-CPC was associated with a higher incidence of early postoperative inflammation and transient visual disturbance, whereas UCP demonstrated a more gradual IOP-lowering trajectory and fewer severe adverse events (8). These differences may have biomechanical relevance, as excessive postoperative inflammation and scleral injury have been implicated in long-term alterations of ocular rigidity and structural integrity.

### 5.5 Glaucoma subtype–specific considerations: PACG versus POAG

Primary angle-closure glaucoma (PACG) and primary open-angle glaucoma (POAG) differ fundamentally in baseline anterior segment configuration and biomechanical loading conditions, factors that may influence structural responses following UCP (20). PACG eyes typically exhibit shallower anterior chambers, thicker lenses, and altered iridocorneal relationships, whereas POAG eyes generally maintain relatively preserved anterior chamber anatomy but experience chronic pressure-related biomechanical stress on the sclera and lamina cribrosa.

Studies in mixed or POAG-predominant cohorts indicate that higher baseline IOP and advanced disease stage may correlate with reduced long-term success, underscoring the role of chronic biomechanical loading in modulating treatment response. Although direct biomechanical comparisons between PACG and POAG eyes after UCP are currently lacking, available evidence does not suggest that UCP exacerbates subtype-specific anatomical vulnerabilities. Future investigations incorporating anterior segment imaging and quantitative biomechanical assessments across glaucoma subtypes are needed to clarify whether pressure-driven versus anatomy-driven mechanisms differentially shape postoperative outcomes.

### 5.6 Special considerations in pediatric eyes

Pediatric eyes possess distinct biomechanical characteristics, including lower scleral rigidity, higher tissue elasticity, and ongoing ocular growth, which collectively render them more deformable under intraocular pressure fluctuations and external energy-based interventions. Experimental and clinical observations summarized by Wójcik-Niklewska highlight that reduced collagen cross-linking density and immature scleral architecture in children contribute to increased compliance and susceptibility to long-term shape remodeling when exposed to sustained biomechanical stress (30).

Direct clinical data on UCP in pediatric glaucoma remain extremely limited; therefore, insights must be extrapolated from related non-incisional cyclodestructive techniques. Recent studies evaluating MP-CPC in pediatric populations have reported clinically meaningful IOP reductions, often exceeding 30%, with acceptable short-term safety profiles and limited severe complications (31). These findings suggest that controlled, non-incisional modulation of ciliary body function can be tolerated in developing eyes. At present, UCP should be applied cautiously in children, with careful patient selection and rigorous long-term structural monitoring, and pediatric-specific studies are clearly warranted.

## 6 Clinical implications

### 6.1 Postoperative monitoring of anterior segment and IOP changes

Prospective clinical series consistently report significant IOP reduction as early as 6 months after UCP, with good tolerability and sustained pressure control (32). Although detailed imaging endpoints are not universally reported, transient anterior chamber deepening or angle widening is occasionally observed, likely reflecting early biomechanical adaptation to reduced IOP. These findings underscore the importance of structured postoperative monitoring, including early and intermediate IOP assessment and anterior segment evaluation, particularly in eyes with advanced disease or prior surgery.

### 6.2 Refractive stability and short-term visual fluctuations

Refractive and visual outcomes following UCP are largely stable across clinical cohorts. In a large BMC Ophthalmology series, 40–60% of eyes showed unchanged BCVA at 18 months, and visual decline, when present, was most often attributable to cataract progression or pre-existing fundus pathology rather than UCP itself (33).

Although dedicated refractive studies remain limited, available evidence suggests that modest early increases in corneal astigmatism typically stabilize within 3–6 months, with visual acuity returning toward baseline (6). These observations support counseling strategies that emphasize transient early visual fluctuations while reassuring patients regarding long-term refractive stability.

### 6.3 Integration of UCP into multimodal glaucoma care

Given its favorable safety and IOP-lowering profile, UCP may be incorporated into broader glaucoma management strategies for selected patients. The 3-year refractory glaucoma study described above, with sustained IOP reduction and few serious complications, supports UCP as a valid alternative to traditional cyclodestructive procedures or as an option when incisional surgery is high risk or contraindicated (3).

Prospective data further indicate that UCP is effective as a salvage intervention after failed glaucoma surgery, achieving IOP reductions approaching 50% at 12 months (19). Comparative cohorts suggest efficacy comparable to other energy-based ciliary procedures, often with fewer severe adverse events.

### 6.4 Transient adverse effects and practical management

Clinical evidence consistently indicates that adverse effects following UCP are predominantly mild and transient. Common findings include conjunctival hyperemia, mild anterior chamber inflammation, and transient hypotony, all resolving with conservative management (33). In a representative cohort of 61 eyes, postoperative conjunctival hyperemia resolved within 1 week to 1 month, mild anterior chamber inflammation subsided within 1–2 weeks, and occasional transient hypotony (IOP ≈ 5 mmHg) was effectively managed by temporary cessation of IOP-lowering medications; notably, a single case of choroidal detachment responded well to systemic and topical steroids combined with cycloplegics. These observations highlight that postoperative care after UCP should emphasize early anti-inflammatory therapy (topical steroids or NSAIDs), close short-term IOP monitoring to detect hypotony or pressure spikes, and prompt use of cycloplegics and steroids when choroidal effusion is suspected. Equally important is patient counseling to differentiate expected, self-limited postoperative symptoms from warning signs that warrant urgent evaluation. Across available prospective and retrospective cohorts, major vision-threatening complications, including phthisis bulbi, are exceedingly rare, underscoring the favorable safety and tolerability profile of UCP when accompanied by structured postoperative management.

## 7 Limitations and future directions

Despite increasing clinical use, evidence regarding UCP’s biomechanical and refractive effects remains limited. Most studies emphasize IOP and safety outcomes, with sparse inclusion of standardized biomechanical metrics such as corneal deformation response, hysteresis, or scleral stiffness (8). Heterogeneity in study design and short- to medium-term follow-up further restricts mechanistic insight (34).

Future research should prioritize prospective, standardized studies integrating biomechanical, structural, and refractive assessments, incorporating advanced imaging and elastography techniques to refine patient selection and improve risk stratification (35).

## 8 Conclusion

UCP represents a valuable minimally invasive option for glaucoma management, offering effective IOP reduction with a generally favorable safety profile. Although transient anterior segment and refractive changes may occur, these effects are typically mild, self-limited, and clinically manageable. The lack of direct *in vivo* biomechanical measurements remains a key knowledge gap, and future studies incorporating advanced biomechanical assessment tools are essential to fully elucidate how UCP reshapes the ocular mechanical environment.
